# Functional Oral Intervention Associated With Reduced Dental Calculus Formation and Secondary Facial Changes: A Case Report

**DOI:** 10.7759/cureus.105231

**Published:** 2026-03-14

**Authors:** Tomomi Ueda

**Affiliations:** 1 Department of Dental Hygiene, Sugimoto Dental Clinic, Kyotanabe, JPN

**Keywords:** case report, dental calculus, facial morphology, mask mouth, myofunctional therapy, oral function

## Abstract

Dental calculus accumulation is traditionally attributed to inadequate oral hygiene; however, oral function may also influence its formation. This case report describes a patient who presented with a sudden increase in calculus formation on the lingual surface of mandibular anterior teeth, despite no changes in oral hygiene habits. Clinical assessment revealed lip incompetence and signs suggestive of mouth breathing following prolonged mask use. A functional oral intervention, focusing on tongue mobility, bilateral mastication, and lip competence, was implemented without modifying brushing instructions. After six months, calculus deposition significantly decreased, returning to a minimal granular pattern, and improvements in lip competence and facial appearance were observed. This case suggests that oral function may influence patterns of dental calculus formation.

## Introduction

Dental calculus formation is commonly regarded as a consequence of plaque retention related to inadequate oral hygiene. However, the intraoral environment is dynamic and influenced by oral functions such as respiration, mastication, and tongue posture, which affect salivary flow and clearance [1,2]. Normally, the movement of the tongue and lips plays a crucial role in the self-cleansing mechanism of the oral cavity. When these functions are compromised - for example, due to mouth breathing or low tongue posture - salivary flow may be altered, potentially leading to stagnation and mineral precipitation.

During the COVID-19 pandemic, prolonged mask use has been associated with increased mouth breathing and oral discomfort [3]. Despite the clinical relevance of these functional factors, traditional preventive dentistry often focuses solely on mechanical cleaning (brushing and scaling), overlooking the impact of oral function. This case report describes a patient in whom a sudden increase in dental calculus formation occurred without changes in oral hygiene practices, and was subsequently reduced following functional oral intervention. This report aims to highlight the potential link between oral function and calculus deposition patterns.

## Case presentation

A female patient with a history of stable oral health and minimal calculus formation during regular six-month recall visits presented with a complaint of increased calculus accumulation on the lingual surface of the mandibular anterior teeth. The patient reported no changes in diet, medication use, systemic health, or oral hygiene routine. Although biochemical blood tests were not performed at this private dental clinic, the patient undergoes mandatory annual health screenings as required by Japanese law, and no systemic abnormalities or metabolic disorders have been reported.

Clinical findings (baseline)

Intraoral examination revealed moderate, sheet-like calculus deposits, confined primarily to the cervical region of the mandibular anterior lingual surfaces. Extraoral assessment showed lip incompetence at rest and features suggestive of mouth breathing. Assessment was based on clinical observation and standardized intraoral photography, compared with historical control images from previous maintenance visits.

Outcomes

The intervention focused on improving oral function through three primary approaches. First, tongue mobility exercises were introduced, consisting of water swishing performed once daily, with 40 repetitions directed upward, downward, and laterally. Second, masticatory function was addressed by encouraging bilateral chewing at every meal to ensure balanced muscle usage. Third, lip competence was targeted through daytime lip-seal practice. During the early phase of treatment, vertical lip taping during sleep was also recommended as a temporary supportive measure to promote nasal breathing.

**Figure 1:**
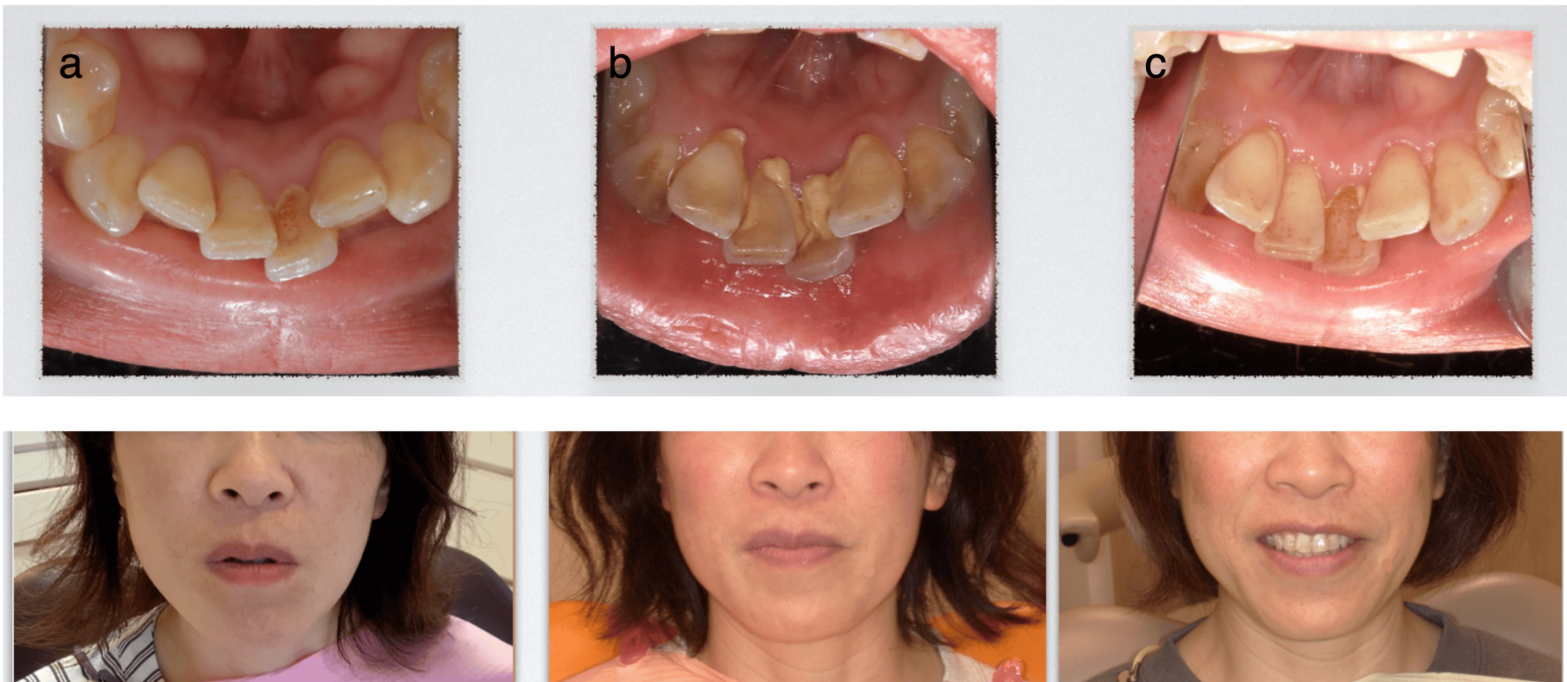
Chronological changes in calculus and facial features: historical control, baseline, and post-intervention. (a) Historical appearance at previous recalls, showing minimal calculus (control). (b) Baseline presentation, showing a sudden increase in calculus formation and lip incompetence. (c) Post-intervention status at the six-month follow-up showing a significant reduction in calculus and improved lip competence. Note: The upper row shows the intraoral view of the mandibular anterior teeth, and the lower row shows the extraoral view of the lower face. The patient provided written and signed consent, allowing publication of this identifiable facial image in an open-access journal.

At the six-month follow-up, calculus deposition on the mandibular anterior lingual surfaces was reduced to a thin, band-like formation, despite no change in oral hygiene practices (Figure 1). The patient was followed up for 13 months, with no interruptions in maintenance visits. Assessment was based on clinical observation and standardized intraoral photography.

Lip competence at rest improved, and the patient reported reduced oral dryness. Secondary improvements in facial appearance were also observed.

## Discussion

Although biochemical blood tests or gallbladder ultrasound were not performed at our dental clinic, the patient undergoes mandatory annual health screenings, as required by Japanese law, with no systemic abnormalities reported. Furthermore, the patient's body weight and lifestyle remained stable during the observation period, suggesting that the changes were primarily functional.

This case challenges the conventional view that dental calculus formation is solely related to oral hygiene practices. In the present case, calculus accumulation increased abruptly, without changes in mechanical cleaning habits, suggesting the involvement of functional factors.

The mandibular anterior lingual region is continuously bathed in saliva from the submandibular and sublingual glands. Normally, the movement of the tongue tip provides a natural self-cleansing action that disperses saliva and prevents mineral precipitation. However, when oral function is compromised - such as through low tongue posture associated with mouth breathing - this cleansing mechanism is lost.

We hypothesize that the calculus formation in this case was driven by salivary stagnation, resulting from functional impairment. The lack of proper tongue movement created a "void" where saliva could pool and stagnate. This stagnation promotes the supersaturation and precipitation of calcium and phosphate ions, leading to rapid calculus deposition. Thus, the calculus observed was likely a physical marker of this functional stagnation, rather than a chemical consequence of evaporation or poor hygiene alone.

Our hypothesis is supported by existing literature linking oral function to intraoral health. For instance, Wagaiyu and Ashley demonstrated that nasal obstruction and mouth breathing are significantly associated with gingival inflammation, suggesting that airflow patterns directly alter the oral environment [2]. Furthermore, Matsui et al. highlighted the importance of oral hypofunction, particularly reduced tongue pressure, in elderly populations, noting its impact on overall oral cleanliness [3]. While these studies focus on gingivitis or general hygiene, our case extends these findings by identifying specific calculus deposition patterns as a potential early marker of such functional deficits, specifically salivary stagnation caused by low tongue posture.

Functional intervention, targeting tongue mobility, mastication, and lip competence, was associated with reduced calculus formation in this patient, likely by restoring the natural clearance mechanism. Secondary improvements in facial appearance were also noted; however, causality cannot be established from a single case. These findings are consistent with principles of orofacial myology, emphasizing the role of muscular coordination in oral health. Overall, this case suggests that patterns of dental calculus deposition may reflect functional impairment, rather than oral hygiene status alone [4].

## Conclusions

This case highlights the critical link between oral function and the intraoral environment. The observed reduction in dental calculus following functional intervention supports the hypothesis that salivary stagnation, driven by impaired tongue mobility and mouth breathing, can precipitate calculus formation, even in the presence of standard oral hygiene. Dental professionals should consider functional factors, such as tongue posture and lip competence, when evaluating patients with unexplained calculus deposition. Addressing these underlying functional deficits may provide a non-invasive and effective pathway for improving both oral cleanliness and facial balance.
